# In vitro antioxidant activity of *Ficus carica* L. latex from 18 different cultivars

**DOI:** 10.1038/s41598-020-67765-1

**Published:** 2020-07-02

**Authors:** M. Shahinuzzaman, Zahira Yaakob, Farah Hannan Anuar, Parul Akhtar, N. H. A. Kadir, A. K. Mahmud Hasan, K. Sobayel, Majid Nour, Hatem Sindi, Nowshad Amin, K. Sopian, Md. Akhtaruzzaman

**Affiliations:** 1 Department of Chemical Sciences, Faculty of Science and Technology, Universiti Kebangsaan Malaysia, 43600 Bangi, Selangor Malaysia; 2 Department of Chemical and Process Engineering, Faculty of Engineering and Built Environment, Universiti Kebangsaaan Malaysia, UKM, 43600 Bangi, Selangor Malaysia; 3 School of Fundamental Science, Universiti Malaysia Terengganu, Terengganu, Malaysia; 4 Solar Energy Research Institute, Universiti Kebangsaan Malaysia, 43600 Bangi, Selangor Malaysia; 5 Department of Electrical and Computer Engineering, Faculty of Engineering, King Abdulaziz University, 21589 Jeddah, Saudi Arabia; 6 Institute of Sustainable Energy, Universiti Tenaga Nasional (@The National Energy University), Jalan IKRAM-UNITEN, 43000 Kajang, Selangor Malaysia; 7 Graduate School of Pure and Applied Sciences, University of Tsukuba, Tsukuba, Ibaraki 305-8573 Japan

**Keywords:** Cancer, Chemical biology, Drug discovery, Plant sciences

## Abstract

As synthetic antioxidants that are widely used in foods are known to cause detrimental health effects, studies on natural additives as potential antioxidants are becoming increasingly important. In this work, the total phenolic content (TPC) and antioxidant activity of *Ficus carica* Linn latex from 18 cultivars were investigated. The TPC of latex was calculated using the Folin–Ciocalteu assay. 1,1-Diphenyl-2-picrylhydrazyl (DPPH), 2,2′-azinobis-(3-ethylbenzothiazoline-6-sulfonic acid) (ABTS) and ferric ion reducing antioxidant power (FRAP) were used for antioxidant activity assessment. The bioactive compounds from *F. carica* latex were extracted via maceration and ultrasound-assisted extraction (UAE) with 75% ethanol as solvent. Under the same extraction conditions, the latex of cultivar ‘White Genoa’ showed the highest antioxidant activity of 65.91% ± 1.73% and 61.07% ± 1.65% in DPPH, 98.96% ± 1.06% and 83.04% ± 2.16% in ABTS, and 27.08 ± 0.34 and 24.94 ± 0.84 mg TE/g latex in FRAP assay via maceration and UAE, respectively. The TPC of ‘White Genoa’ was 315.26 ± 6.14 and 298.52 ± 9.20 µg GAE/mL via the two extraction methods, respectively. The overall results of this work showed that *F. carica* latex is a potential natural source of antioxidants. This finding is useful for further advancements in the fields of food supplements, food additives and drug synthesis in the future.

## Introduction

Nature is an essential source of substances for human needs. Most of the pharmacological substances and active compounds used to combat various diseases or to prepare drugs are extracted from natural sources. Synthetic antioxidants used widely in food and medicine cause or promote negative health effects. Thus, research on natural additives as potential antioxidants is receiving growing interest. Polyphenolic compounds are important for the human body and can act as antioxidants and free radical scavengers. Therefore, research on various polyphenols from natural resources has now gained considerable attention.^1^ The richest source of natural drugs includes plants (e.g. paclitaxel from *Taxus brevifolia*)^2^ or microorganisms (e.g. penicillin from *Penicillium notatum*)^3^. In this regard, *Ficus carica* is a strong candidate because it is a natural source of polyphenols and bioactive metabolites.

*F. carica*, also known as ‘fig’, is a member of the genus *Ficus* and valued for its fresh and dried fruits^4,5^. The fruits of *F. carica* are an abundant source of vitamins, carbohydrates, minerals, sugars, phenolic compounds and organic acids^6,7^. All of its parts, such as fruits, leaves, shoots, roots and latex, are used to treat various human diseases. The latex of fig shows antioxidant, antifungal, chitinolytic, milk clotting^8^, cytotoxic and antiviral^9,10^, antibacterial^11^ and anthelmintic activities^12,13^. Various bioactive compounds are present in the *F. carica* plant latex. The milky sap from several parts of *F. carica* has been investigated, and two important phenolic compounds, namely, psoralen and bergapten, have been identified. These compounds are more abundant in the leaf sap than in other parts of *F. carica*^14^. Oliveira et al. analysed the latex of *F. carica* and identified 38 bioactive compounds by using gas chromatography mass spectrometry. Seven of the bioactive compounds are phytosterols, 13 are free amino acids, and 18 are fatty acids. They identified phytosterols, such as β-sitosterol, lupeol, α- and β-amyrin, betulol and lanosterol, and amino acids, such as leucine, phenylalanine, tryptophan, histidine, alanine, glutamine, glycine, serine, ornithine, lysine, asparagine, tyrosine and cysteine^15^. Although many researchers have successfully determined the antioxidant activity and total phenolic content (TPC) of crude extracts from *F. carica* leaves, fruits and bark, limited reports on the antioxidant activity and TPC of *F. carica* latex are available. Moreover, the data of published reports are only for few cultivars, and the methods used are complex.

The different methods of extraction include maceration extraction^16^, microwave-assisted extraction^17–19^ and supercritical fluid extraction^20–22^. Most of these approaches, however, are time consuming and require comparatively more solvents than others and are not economically viable given their high cost^23^. However, as a better alternative to these methods, ultrasound-assisted extraction (UAE) is more efficient; requires relatively less solvents; and has good reproducibility, rapid extraction time, low temperature and easy scaling up for application in industries^24–26^. This process breaks down the cell walls, enables the cell content to be washed out and has high efficiency for isolating antioxidant and phenolic compounds^27,28^. Maceration is also a simple, convenient and less costly extraction process in terms of instrumentation^29^. Therefore, this method is more appropriate than others for both small and medium-sized enterprises in developing countries^30^.

Numerous in vitro assays are used to determine the antioxidant activity of biological samples. Comparing one assay with another is hard, and evaluating the antioxidant activity using a single antioxidant test method only is not possible because different methods measure antioxidant activity from different angles^31,32^. Amongst the various in vitro methods, 1,1-diphenyl-2-picrylhydrazyl (DPPH) assay is more simple, rapid and inexpensive, whilst the 2,2′-azinobis-(3-ethylbenzothiazoline-6-sulfonic acid) (ABTS) free radical assay is appropriate for both hydrophilic and lipophilic samples^31^. In this study, the TPC and antioxidant activities of fig latex from 18 different cultivars were evaluated. The Folin–Ciocalteu (FC) assay for TPC and three different in vitro assays, such as DPPH, ABTS and ferric ion reducing antioxidant power (FRAP), were used to determine the antioxidant activities of the samples. Two different extraction methods, such as maceration and UAE, were used. Initially, the solvent effect and the effect of solvent-to-latex ratio were also studied to select the proper solvent for extraction in this study.

## Materials and methods

### Chemicals and reagents

All chemicals were analytical reagent grade and used without further purification. All chemicals with their chemical formulas, manufacturers and purity are listed in Table 1. Milli-Q water was used to prepare standard materials and reactant solutions and perform extraction.

**Table 1:** List of required chemicals used in this research.

Chemicals and reagents	Chemical formula	Purpose	Company	Purity (%)
Methanol	CH_3_OH	Extraction solvent	Friendemann Schmidt, Australia	99.8
Ethanol	C_2_H_5_OH	Extraction solvent	Friendemann Schmidt, Australia	99.9
Ethyl acetate	C_4_H_8_O_2_	Extraction solvent	Friendemann Schmidt, Australia	99.5
*n*-Hexane	C_6_H_14_	Extraction solvent	Sigma-Aldrich (USA)	99
DPPH	C_18_H_12_N_5_O_6_	DPPH assay	Sigma-Aldrich (USA)	95
ABTS	C_18_H_18_N_4_O_6_S_4_	ABTS assay	Sigma-Aldrich (USA)	98
2,4,6-tri(2-pyridyl)-s-triazine	C_18_H_12_N_6_	FRAP assay	Sigma-Aldrich (USA)	99
Ferric chloride	FeCl_3_	FRAP assay	Sigma-Aldrich (USA)	97
6-Hydroxy-2,5,7,8-tetramethylchroman-2-carboxylic acid (Trolox)	C_14_H_18_O_4_	Standard	Sigma-Aldrich (USA)	97
FC reagent	C_6_H_6_O	TPC analysis	Merck Millipore (Germany)	–
Sodium carbonate	Na_2_CO_3_	TPC analysis	Merck Millipore (Germany)	99.8
Potassium persulfate	K_2_S_2_O_8_	FRAP assay	Friendemann Schmidt, Australia	99
Potassium ferricyanide	C_6_N_6_FeK_3_	FRAP assay	Sigma-Aldrich (USA)	99.5
Trichloroacetic acid	C_2_HCl_3_O_2_	FRAP assay	Friendemann Schmidt, Australia	99.5
Monohydrate gallic acid	C_7_H_8_O_6_	Standard for TPC	Friendemann Schmidt, Australia	99
Hydrochloric acid	HCl	FRAP assay	Friendemann Schmidt, Australia	37

### Equipment

The equipment used in this study and their manufacturers with model number are listed in Table 2. An orbital shaker, Thermoline ultrasonic bath, centrifuge machine and steam distillatory were used for extraction. A UV–Vis spectrophotometer was used to determine the antioxidant capacities of the samples.

**Table 2:** Equipment used in this study.

Equipment	Model/company
Incubator shaker	HY-5A, Zihe International Trade (Shanghai) Co., Ltd, China
Thermoline ultrasonic bath	220 V and 40 kHz, Zihe International Trade (Shanghai) Co. Ltd., China
Centrifuge machine	80-2B, 220 V 50 Hz, Zihe International Trade (Shanghai) Co. Ltd., China
UV–Vis spectrophotometer	756 PC, Shanghai Yuefeng Instruments & Meters Co., Ltd

### Latex sample collection and preparation

**Figure 1:**
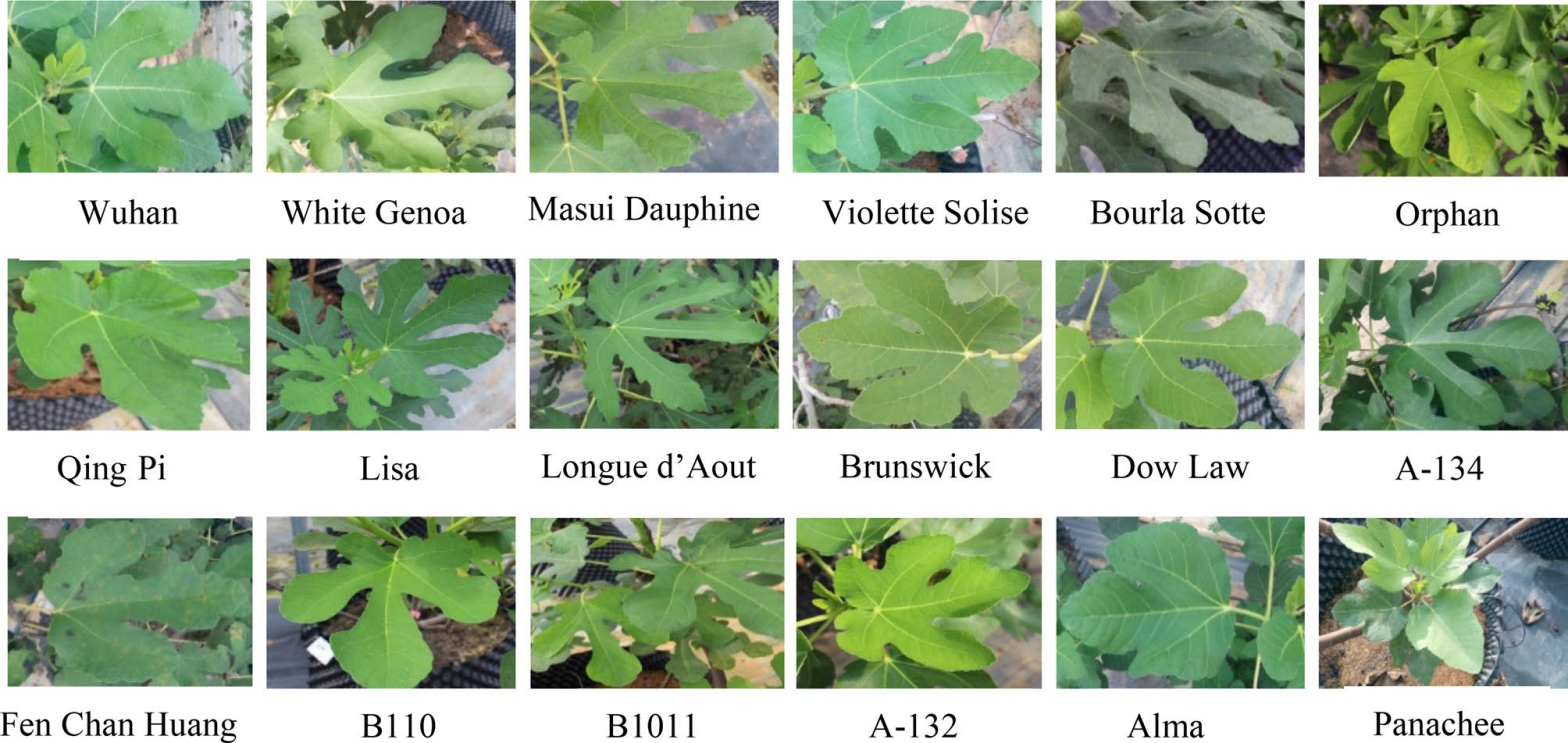
Leaves from the 18 cultivars of *F. carica.*

The latex samples from 18 different cultivars of *F. carica* were collected during daytime from March to June 2018 from Saf Fa Fig Garden in the Living laboratory Energy and Future Crops Laboratories at Kuala Pilah, under the Faculty of Engineering and Built Environment, Universiti Kebangsaan Malaysia, Bangi, Selangor, Malaysia. The *F. carica* cultivars were identified and imported from the Forest Research Institute of Liaoning, China. Latex was collected manually from three-and-a-half-year-old *F. carica* plant leaf shoot. The shoot was manually wrecked, and the latex was collected drop by drop into a 15 mL glass vial without pressing. The latex was homogenised, weighted, aliquoted and analysed. Figure 1 shows the green leaves from the 18 cultivars of *F. carica*.

### Extraction of crude sample from *F. carica* latex

Various methods are used to extract antioxidant compounds from plant materials. In this study, modified maceration extraction with continuous shaking and UAE were used with the same conditions. The extraction conditions were selected on the basis of the primary screening and optimisation of this study mentioned in our previous work^23^.

For maceration extraction, the samples were extracted using an incubator shaker at 200 rpm and 35 °C. Ultrasonication was conducted by using a Thermoline ultrasonic bath at 35 °C. *F. carica* latex (1 g) from the cultivar ‘Wuhan’ was kept in two different 25 mL capped long glass vial, and 10 mL of 75% ethanol was added in each vial. Then, the mixtures were transferred into the shaker and ultrasonic bath for maceration and ultrasonication for 30 min. After extraction, the samples were centrifuged at 4,000 rpm for 10 min by using a laboratory centrifuge machine. The supernatant liquids were filtered and used to determine TPC and antioxidant activity and to perform other analyses. The same extraction process was repeated for the cultivars ‘White Genoa’, ‘Masui Dauphine’, ‘Violette Solise’, ‘Bourla Sotte’, ‘Orphan’, ‘Qing Pi’, ‘Lisa’, ‘Longue d’Aout’, ‘Brunswick’, ‘Dow Law’, ‘A-134’, ‘Fen Chan Huang’, ‘B110’, ‘B1011’, ‘A-132’, ‘Alma’ and ‘Panachee’ successively.

### Effect of solvent

The solvent effects were investigated via maceration extraction for the cultivar ‘White Genoa’ before the final extraction of all cultivars. From the primary screening data of this study, ‘White Genoa’ extract obtained via maceration showed the highest TPC and antioxidant activity. Therefore, ‘White Genoa’ was used to investigate the effects of solvent and latex-to-solvent ratio on extraction. Initially, different types of solvents, such as 100% methanol, 100% ethanol, 75% ethanol, 100% ethyl acetate and 100% *n*-hexane, were used with the same extraction condition to investigate the solvent type. ‘White Genoa’ was extracted via maceration, and its TPC and antioxidant activity were determined via DPPH assay to study the effects of solvent. The effect of latex-to-solvent ratio was investigated with four different ratios, such as 1:1, 1:5, 1:10 and 1:15 g/mL (w/v). *F. carica* latex (1 g) from ‘White Genoa’ was extracted using different amounts of 75% ethanol, such as 1, 5, 10 and 15 mL. The result was validated using the same experiment on ‘B110’ as the second highest active cultivar, and the results are shown in the supplementary data (Tables S1 and S2).

### Determination of TPC

The TPC of leaves of *F. carica* was analysed using FC reagent with some modifications^23,33^. The FC reagent was used as the oxidising agent. Standard gallic acid or plant extract (100 μL) was mixed with 3.25 mL of 12 times pre-diluted FC reagent. After proper mixing, the samples were allowed to stand for 7 min; then 750 µL of 20% Na_2_CO_3_ was added to the solution and kept for 2 h in incubation in the dark. Finally, absorbance was recorded at 760 nm on the basis of a colorimetric redox reaction from a standard curve (y = 0.0033x + 0.0471, R^2^ = 0.9951) and using standard gallic acid solution of 31.25–500 µg/mL. The data are shown as μg gallic acid equivalent/mL sample. Each sample was measured as triplicate.

### Determination of antioxidant activity

In this study, three different scavenging assays were used to determine the antioxidant activity from *F. carica* latex. DPPH, ABTS and FRAP assays were used with the same latex samples.

#### DPPH free radical scavenging assay

Antiradical activity was determined spectrophotometrically using a UV–visible spectrophotometer by monitoring the disappearance of DPPH^·^ at 520 nm in accordance with a previously described procedure with some modifications. The reaction mixtures in the sample consisted of 100 μL of supernatant and 3.9 mL of 0.1 mM DPPH^·^ dissolved in ethanol. The samples were incubated for 30 min at room temperature. Every sample was measured in triplicate. Ethanol was used as blank, and the sample without antioxidant was used as control. Trolox equivalent antioxidant capacity (TEAC) was calculated by preparing a standard Trolox curve (y = -0.0008x + 0.4956, R^2^ = 0.9998) from 31.25 µg/mL to 1.0 mg/mL of a standard Trolox solution. The outcomes were presented as mg Trolox equivalent (TE)/g sample. Each experiment was carried out in triplicate. The DPPH activity was expressed as a percentage of inhibition and calculated using Eq. (1) ^34^:

1 $$\% {\text{ Inhibition}} = \, (1 - A_{S} /A_{B} ) \times 100\%$$

where *A*_*B*_ = absorbance of control sample (*t* = 0 h) and *A*_*S*_ = absorbance of a tested sample after the reaction (*t* = 1 h).

#### ABTS radical scavenging assay

The ABTS radical scavenging assay was calculated on the basis of the method of Gorinstein^35^ with some modifications. Firstly, the radical solution was prepared by mixing stock solutions, such as 7 mM aqueous solution of ABTS and 2.45 mM potassium persulfate (K_2_S_2_O_8_) solution at a ratio of 1:1^36^. The mixture was kept for 12–16 h in dark conditions at room temperature. Then, the fresh working solution was prepared for each bioassay by diluting 1 mL of ABTS radical solution with the required amount of ethanol to obtain the absorbance of 0.700 ± 0.02 units at 745 nm. Afterwards, 100 μL of different extracts or different standard Trolox solutions were added to 3.9 mL of an ABTS^+^ solution. The absorbance was measured immediately at 745 nm after 6 min incubation at room temperature. Aqueous ethanol (75%) and Trolox were used as blank and positive control, respectively. TEAC was calculated by preparing a Trolox curve for ABTS assay (the standard curve equation: y = − 0.0009x + 0.4836, R^2^ = 0.9978 from 31.25 µg/mL to 500 µg/mL), and the results were presented as μg TE/mL sample. The percentages of inhibition of ABTS was calculated using Eq. (1).

#### FRAP assay

The FRAP of fig latex was determined using the potassium ferricyanide–ferric chloride method described by Oyaizu^37^ with some modifications. The ethanolic extracts (100 μL aliquots) of *F. carica* latex were added to 2.5 mL of phosphate buffer (0.2 M, pH 6.6) and 2.5 mL of potassium ferricyanide (1%). After 20 min of incubation at 50 °C of the mixtures, 2.5 mL of trichloroacetic acid (10%) was added. From the mixture, 2.5 mL was taken and again mixed with 2.5 mL of water and 0.5 mL of 1% FeCl_3_. The absorbance of the mixture was measured at 593 nm after 30 min of allowing the solution to stand. The results were expressed as TEAC in mM/L Trolox. TEAC was calculated by preparing a Trolox curve for FRAP assay (the standard curve equation: y = 0.0007x + 0.0645, R^2^ = 0.9998) from 31.25 µg/mL to 1.0 mg/mL of standard Trolox solution. Each experiment was carried out in triplicate.

### Statistical analysis

To study the variance of antioxidant activity and phenolic content of various cultivars of *F. carica*, data were processed by one-way ANOVA via STATGRAPHICS Centurion XVII (Version 17.2.00, Stat Points Technologies Inc. 1982–2016). Correlation, regression and cluster analyses were carried out in STATGRAPHICS Centurion XVII. Statistically significant differences were determined by Tukey’s honest significant difference (HSD) post hoc test. F values at p < 0.05 were considered statistically significant. Pearson product–moment correlation matrix and regression analysis were used to evaluate the connection amongst DPPH, ABTS, FRAP and TPC in the extraction processes. The data of TEAC and GAE curve were analysed in Microsoft Excel 10 (Microsoft Inc., Redmond, WA, USA). All data were analysed in triplicate and expressed as means ± standard deviation (SD).

## Results

### Effect of solvent type and solvent-to-latex ratio

To obtain better activity of natural extracts, it is very essential to select proper solvents and solvent ratios. Therefore, the solvent type and solvent-to-sample ratio were investigated before extraction as mentioned previously^23^. Methanol and ethanol are the main polar solvents used for extracting antioxidants and TPC from plant materials. Table 3 shows the effect of different solvents on the DPPH and TPC of *F. carica* latex.

**Table 3:** Effects of solvents on the DPPH and TPC of the cultivar ‘White Genoa’ of *F. carica* latex.

Solvent	DPPH (%)		TPC (µg GAE/mL)	
	Maceration	Ultrasonic	Maceration	Ultrasonic
Methanol (CH_3_OH) 100%	66.67 ± 1.30a	60.81 ± 1.92a	354.32 ± 10.45a	332.18 ± 11.69a
Ethanol (C_2_H_5_OH) 100%	52.72 ± 0.96c	48.37 ± 1.28b	274.62 ± 8.26c	268.09 ± 10.11c
Ethanol (C_2_H_5_OH) 75%	63.76 ± 1.48b	59.16 ± 2.05a	298.15 ± 6.59b	291.25 ± 8.71b
Ethyl acetate (C_4_H_8_O_2_) 100%	22.52 ± 0.35d	25.04 ± 0.74c	94.03 ± 4.18d	83.29 ± 2.59d
*n*-Hexane (C_6_H_14_) 100%	11.90 ± 0.20e	10.32 ± 0.31d	62.85 ± 3.27e	59.83 ± 2.08e

Data are represented as the mean ± SD of three measurements. Different letters (a–e) for each column symbolise significant differences (p < 0.05) by means of Tukey’s HSD test.

*F. carica* latex extracted using 100% methanol had a higher activity (DPPH, 66.67%; TPC, 354.32 µg of GAE/mL) than the latex extracted via 100% ethanol (DPPH, 52.72%; TPC, 274.62 µg GAE/mL). However, ethanol was used as the master solvent in this study given the high toxicity of methanol^38–41^. The activity of 75% ethanol (DPPH, 63.76%; TPC, 298.15 µg GAE/mL) was higher than that of 100% ethanol. The effect of latex-to-solvent ratio was also studied with different latex-to-solvent ratios (1/1, 1/5, 1/10 and 1/15 g/mL) over optimum condition (30 °C, 35 min and 75% ethanol). The outcomes are shown in Table 4. The antioxidant activity increased with the increase of solvent up to 5 mL and then decreased as the amount of solvent increased. Antioxidant activity for 5 and 10 mL solvent were nearly the same. The TPC decreased with the increase of solvent up to 15 mL.

**Table 4:** Effect of solvent-to-latex ratio on the DPPH scavenging capacity and TPC of ‘White Genoa’ latex extract obtained via maceration.

Latex-to-solvent ratio (w/v)	DPPH scavenging capacity (%)	TPC (μg GAE/mL)
1:1	65.76 ± 2.17b	340.5 ± 13.30a
1:5	74.48 ± 2.90a	299.62 ± 8.69b
1:10	66.21 ± 3.04b	291.68 ± 10.22b
1:15	51.57 ± 2.11c	234.03 ± 6.91c

Data are presented as the mean ± SD of three measurements. Different letters (a–d) for each column symbolise significant differences (p < 0.05) by means of Tukey’s HSD test.

### TPC and antioxidant activity of 18 cultivars of *F. carica* latex

#### TPC

**Figure 2:**
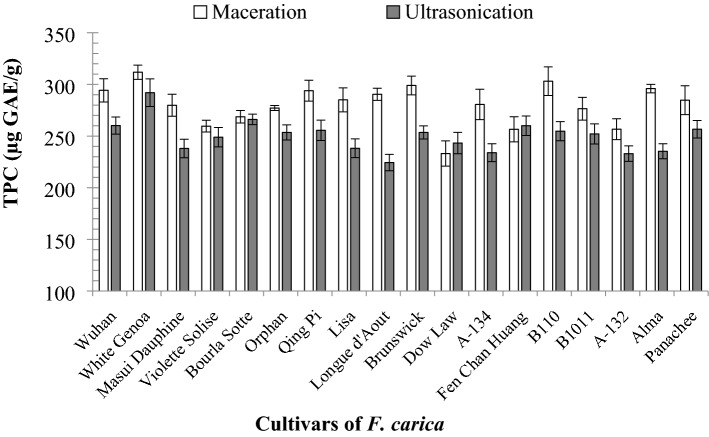
TPC of the 18 *F. carica* cultivar latex extracts via FC assay (data were calculated as the mean ± SD of three measurements represented along with the error bar).

The results of TPC of *F. carica* latex extracted by maceration extraction and ultrasonic extraction are presented in Fig. 2. Amongst all the 18 cultivars, the maceration extraction showed the highest TPC (from 233.14 ± 12.25 µg GAE/mL to 311.83 ± 6.93 µg GAE/mL) compared with UAE (224.37 ± 7.95 µg GAE/mL to 291.98 ± 13.40 µg GAE/mL) (p < 0.050).

Amongst the cultivars extracted via maceration, ‘White Genoa’ showed the highest TPC (311.83 ± 6.93 µg GAE/mL), whereas ‘Dow Law’ showed the lowest activity (233.14 ± 12.25 µg GAE/mL). Amongst the cultivars extracted via UAE, ‘White Genoa’ showed the highest TPC (291.98 ± 13.40 µg GAE/mL), whereas ‘Longue d’Aout’ showed the lowest TPC (224.37 ± 7.95 µg GAE/mL). ‘B110’ was the cultivar with the second highest TPC (303.14 ± 13.80 µg GAE/mL TPC) via maceration extraction. ‘Bourla Sotte’ showed the second highest TPC (266.17 ± 5.04 µg GAE/mL) via UAE.

#### DPPH free radical scavenging activity

**Figure 3:**
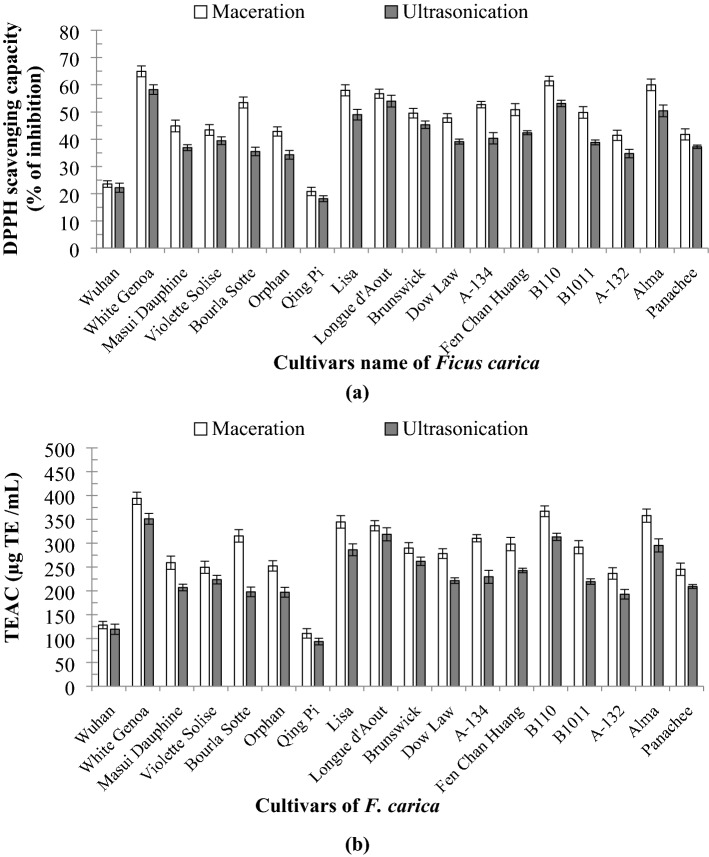
(**a**) DPPH percentage inhibition of the 18 cultivars of *F. carica* latex obtained by maceration and UAE, (**b**) DPPH inhibition in µg TE/mL of the 18 cultivars of *F. carica* latex obtained by maceration and UAE (data were calculated as the mean ± SD of three measurements and represented along with the error bar).

The DPPH scavenging activities of the latex of 18 *F. carica* cultivars were evaluated at the same extraction conditions (30 °C extraction temperature, 35 min extraction time and 75% ethanol as extraction solvent), and the results are presented in Fig. 3a and b. For both extraction methods, the DPPH antioxidant activity was analysed and expressed as percentage inhibition and TEAC. The percentage of DPPH activity for maceration extraction ranged from 20.82% ± 1.54 to 64.93% ± 2.00% and 110.75 ± 9.92 µg to 394.17 ± 12.82 µg TE/mL for percentage of inhibition and TEAC, respectively. The activities of the extracts obtained via UAE ranged from 18.16% ± 1.07 to 58.22% ± 1.78% and 93.67 ± 6.88 µg to 351.08 ± 11.41 µg TE/mL, respectively. Amongst the 18 cultivars, ‘Qing Pi’ showed the lowest antioxidant activity (20.82% ± 1.54% and 110.75 ± 9.92 µg TE/mL), whereas ‘White Genoa’ showed the highest activity (64.93% ± 2.00% and 394.17 ± 12.82 µg TE/mL) via maceration. ‘B110′ showed the second highest DPPH activity, (61.38% ± 1.75% and 367.00 ± 11.25 µg TE/mL). The cultivar ‘Alma’ showed the third highest antioxidant activity (59.97% ± 2.15% and 367.00 ± 11.25 µg TE/mL). For UAE, ‘White Genoa’ showed the highest activity, whereas ‘Qing Pi’ showed the lowest activity. ‘White Genoa’ showed 58.22% ± 1.78% inhibition effect and 351.08 ± 11.41 µg TE/mL TEAC for UAE. The difference amongst ‘Longue d’Aout’, ‘B110’, ‘Alma’ and ‘Lisa’ for the UAE process was not significant.

#### ABTS^+^ radical scavenging activity of* F. carica* latex

**Figure 4:**
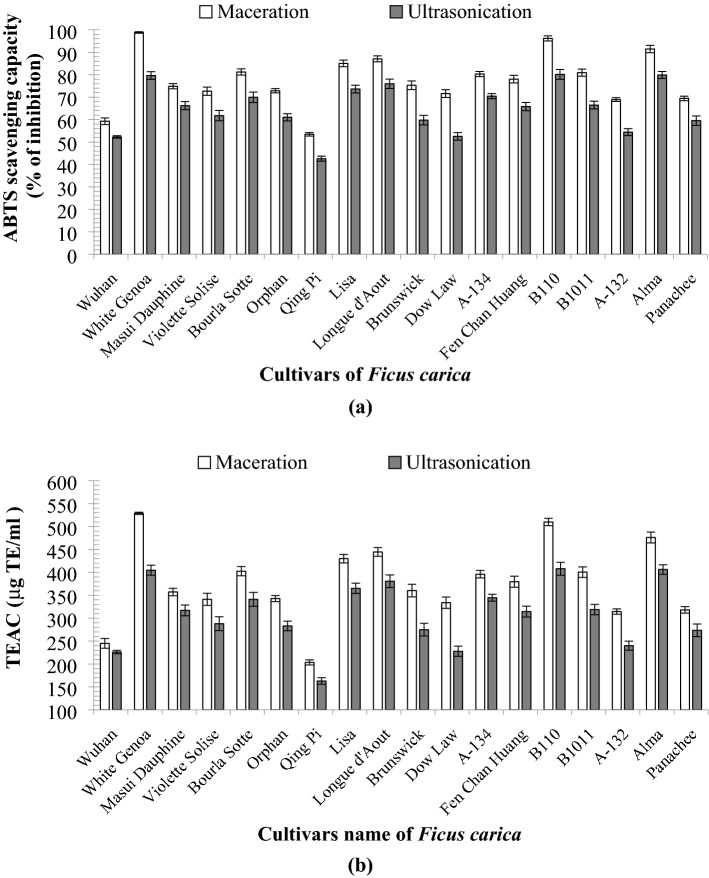
(**a**) ABTS percentage inhibition of 18 cultivars of *F. carica* latex obtained by maceration and UAE, (**b**) ABTS inhibition in µg TE/mL of the 18 cultivars of *F. carica* latex by maceration and UAE (data were calculated as the mean ± SD of three measurements and represented along with the error bar).

The results of the ABTS radical scavenging assay were expressed as the percentage of inhibition and TEAC similar to DPPH and shown in Fig. 4a and b. Amongst the 18 cultivars, ‘White Genoa’ showed the highest activity (98.81% ± 0.34% and 79.64% ± 1.69% inhibition) for maceration and UAE, respectively. The TEAC capacities for ‘White Genoa’ were 528.78 ± 2.44 and 414.55 ± 11.03 µg TE/mL for maceration and UAE, respectively. The latex of ‘B110′ showed 96.18% ± 1.13% and 80.14% ± 2.19% inhibition and 509.90 ± 8.12 µg TE/mL and 407.83 ± 14.27 µg TE/mL TEAC using the two extraction processes, which is the second highest activity amongst the cultivars.

#### Antioxidant activity of* F. carica* latex via FRAP assay

**Figure 5:**
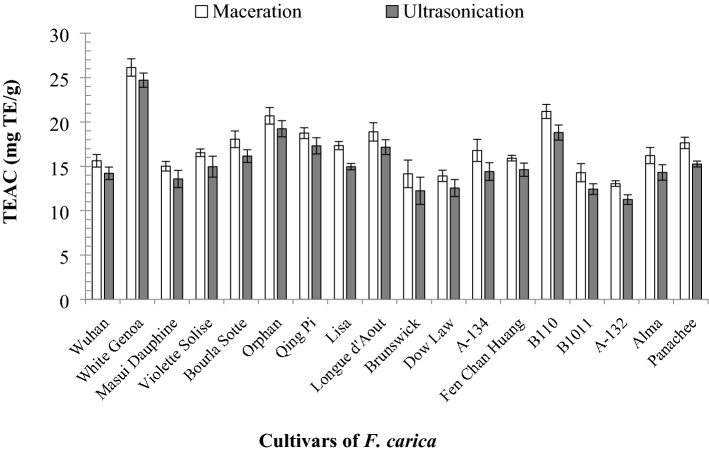
Antioxidant activity in FRAP assay of the 18 cultivars of *F. carica* latex by maceration and UAE (data were calculated as the mean ± SD of three measurements and represented along with the error bar).

The antioxidant activities of the *F. carica* latex obtained by maceration and UAE from 18 cultivars were analysed via FRAP assay and expressed through TEAC (mg TE/g). Figure 5 shows that the extract from ‘White Genoa’ obtained by maceration had the highest FRAP value (26.14 ± 0.98 mg TE/g), whilst that of ‘B110′ showed the second highest activity (21.19 ± 0.80 mg TE/g). The FRAP value for the ‘White Genoa’ extract obtained via UAE was 24.71 ± 0.80 mg TE/g, which was the highest value. The second highest value was observed in the cultivar ‘Orphan’ (19.24 ± 0.91 mg TE/g). In the FRAP assay, the ‘A-132’ cultivar extract obtained via maceration (13.05 ± 0.31 mg TE/g) and UAE (11.25 ± 0.54 mg TE/g) showed the lowest antioxidant activity.

### Cluster analysis of cultivars of *F. carica*

Hierarchical cluster analysis is used to classify the *F. carica* cultivars on the basis of their TPC and antioxidant activities. Ward’s method was used to create the dendrogram, and the similarity between cultivars according to their activities was measured using Euclidean distance. Cultivars with higher TPC and antioxidant activities, as indicated by the DPPH, ABTS and FRAP assays, were placed in the same cluster, whereas cultivars with lower antioxidant and TPC activities were placed in a different cluster.

**Figure 6:**
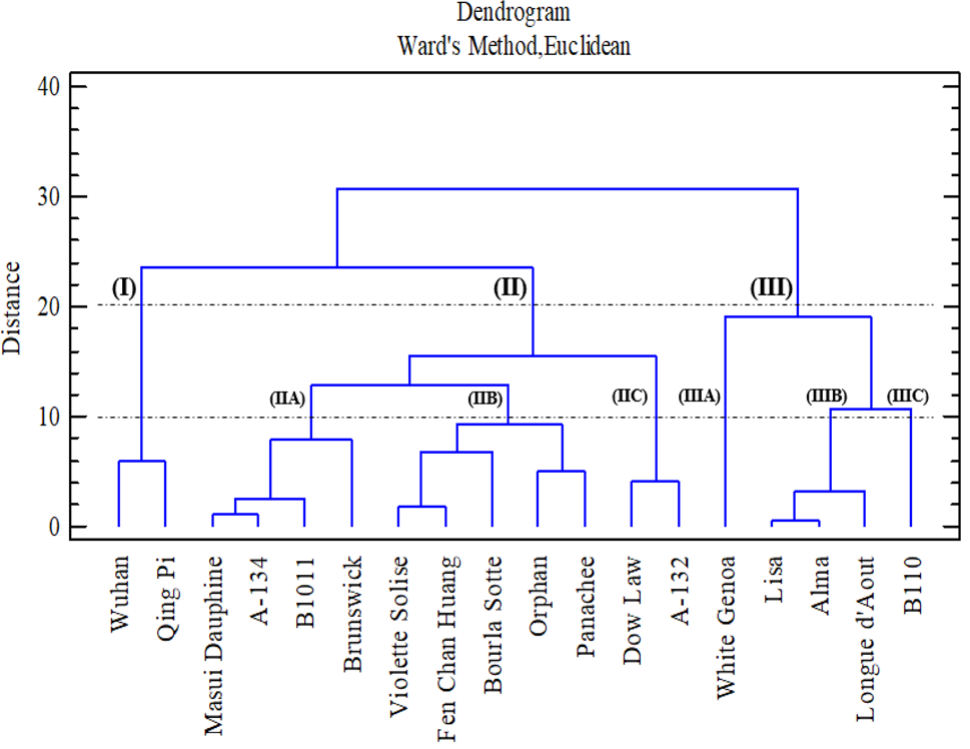
Dendrogram of *F. carica* latex on the basis of antioxidant activity and TPC.

On the basis of antioxidant activity and TPC, three main clusters were obtained at the Euclidean distance of 20.0, including cluster (I), cluster (II) and cluster (III) (Fig. 6). The cultivars in cluster (I) showed the lowest TPC and antioxidant activity. Two cultivars, namely, ‘Wuhan’ and ‘Qing Pi’, were included in this cluster. The second cluster (II), which was the second lowest active cluster based on TPC and antioxidant activity, included 11 cultivars. This cluster included ‘Masui Dauphine’, ‘A-134’, ‘B1011’, ‘Brunswick’, ‘Violette Solise’, ‘Fen Chan Huang’, ‘Bourla Sotte’, ‘Orphan’, ‘Panachee’, ‘Dow Law’ and ‘A-132’. At the Euclidean distance of 10.0, this cluster was divided into three sub-clusters, namely, sub-clusters (IIA), (IIB) and (IIC). According to the linkage distance, sub-cluster (IIB), which was the highest active sub-cluster amongst the three sub-clusters, contained five cultivars, such as ‘Violette Solise’, ‘Fen Chan Huang’, ‘Bourla Sotte’, ‘Orphan’ and ‘Panachee’. Sub-cluster (IIA) was the second highest active sub-cluster with four cultivars (‘Masui Dauphine’, ‘A-134’, ‘B1011’ and ‘Brunswick’); sub-cluster (IIC) (which included ‘Dow Law’ and ‘A-132’) was the least active sub-cluster under cluster (II). Cluster (III) was the most active cluster with five cultivars, including ‘White Genoa’, ‘Lisa’, ‘Alma’, ‘Longue d’Aout’ and ‘B110’. At a Euclidean distance of 10.0, this cluster was also divided into three sub-clusters: (IIIA), (IIIB) and (IIIC). The first sub-cluster (which contained only one cultivar, ‘White Genoa’) had the most active cultivar amongst the three sub-clusters. Sub-clusters (IIIB) and (IIIC) had the least and second least active cultivars, respectively, amongst the three sub-clusters. Sub-cluster (IIIB) included three cultivars, including ‘Lisa’, ‘Alma’ and ‘Longue d’Aout’, whilst sub-cluster (IIIC) included only one cultivar (‘B110’).

### Correlation analysis of *F. carica* latex

**Figure 7:**
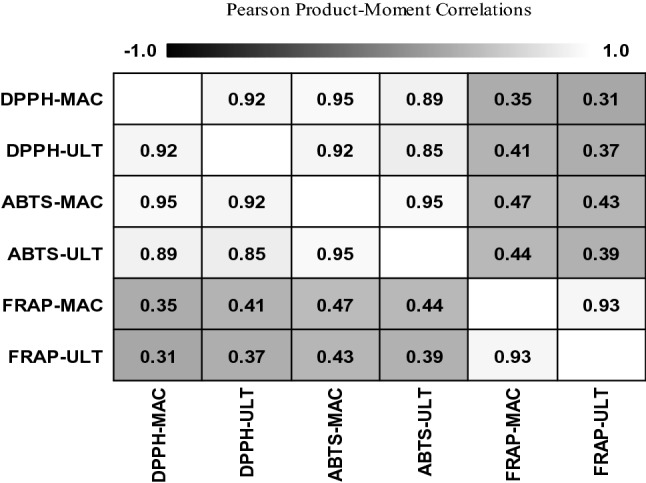
Pearson product–moment correlation matrix of *F. carica* latex.

Significant correlations were obtained amongst the antioxidant activities and TPC via different assays. Figure 7 shows the correlation amongst DPPH, ABTS and FRAP assays of *F. carica* latex extracts obtained via maceration and UAE.

A positive relationship exists between the maceration DPPH and ultrasonic DPPH (r = 0.92). This result indicates a 92% possibility that the same bioactive compounds or the same factors attributed to maceration and UAE influenced the DPPH activity. The DPPH and ABTS activities of extracts obtained via maceration showed a positive correlation with the highest r-value (r = 0.95). So, there have 95% possibility of same reasons, same mechanisms or same bioactive compounds influence the antioxidant activity of *F. carica* latex with DPPH and ABTS assays via maceration. The FRAP assay results indicated a strong positive relationship between maceration and UAE (r = 0.93). However, a very weak but positive correlation was detected amongst FRAP, DPPH and ABTS assays. So, there has a less similarity of mechanism or the compounds which influence the mechanism of FRAP assay compared to the DPPH and ABTS assay.

### Microscopic studies

**Figure 8:**
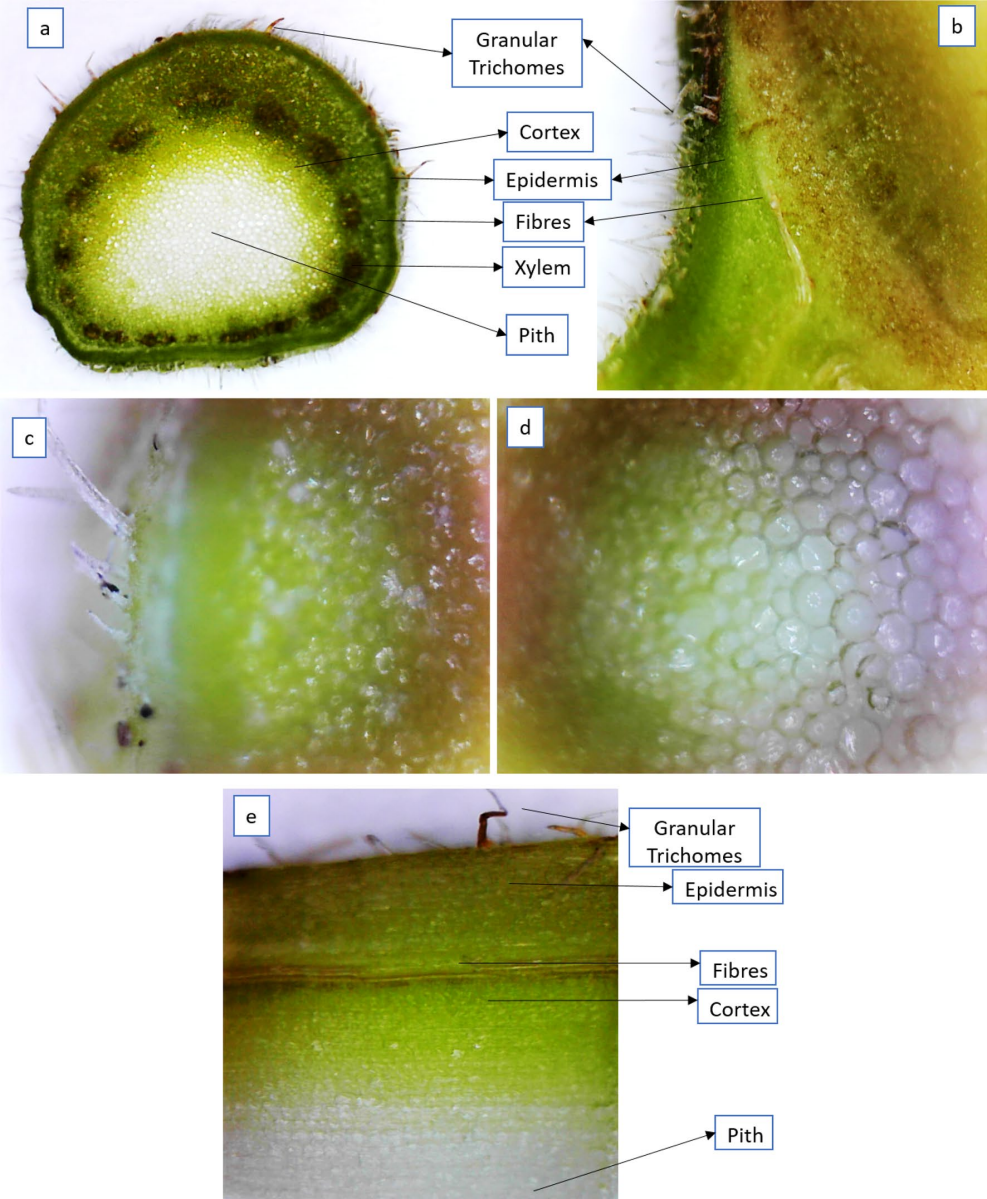
Anatomy of *F. carica* leaf shoot from ‘White Genoa’. (**a**, **b**) Transverse section in 10 × and 20 × projection, (**c**, **d**) 40 × projection, (**e**) Longitudinal section in 20 ×

The structures of leaf anatomy from the leaf shoot of ‘White Genoa’ cultivar was studied under a compound microscope. After chopping, the leaf shoot was cleaned with ethanol, chloroform and acetic acid mixture (60:30:10 v/v) followed by deionised water. Then, the anatomical segment (500 μm) was analysed under the microscope at different projections. Figure 8 shows the transverse section of fig leaf shoot from the ‘White Genoa’ cultivar at 10 × and 20 × projections. The veins inside the lamina are visible to the naked eye. All areal parts of the shoot and the simple hairy granular trichomes can be seen (Fig. 8). The cross-section of petiole showed a number of xylem vessels inside of the fibre, and piths are present in the centre. Small pores can be seen inside of the pith vessels, cortex and fibre of *F. carica* leaf shoot, which may contain the latex (Fig. 8c and d). Figure 8e shows the longitudinal section of fig leaf shoot from the ‘White Genoa’ cultivar.

## Discussion

The biological activity of *F. carica* latex depends on the solvent. A diluted solvent can better extract antioxidants and polyphenols from plants compared with a pure solvent^23,42,43^. Ethanolic extracts of *Psidium guajava* L. have the highest activity amongst chloroform, petroleum ether and water extracts^44^. The bioactive compounds from 70% ethanolic extracts of *Moringa oleifera* show better activity than those of others^30^. The maximum TPC value of the 70% ethanolic extract obtained via maceration was 5.35 g GAE/100 g of powder. Polar solvents are more effective than non-polar solvents in extracting bioactive compounds from plant materials^45^. The current study indicated that non-polar solvents, such as *n*-hexane, and less polar solvents, such as ethyl acetate (polarity index 4.4), showed low capability for extracting bioactive compounds from *F. carica* latex. The activities of the extracts using 1, 5 and 10 mL solvents were significantly different. A high latex-to-solvent ratio increases the rate of diffusion, which improves the solvent-based extraction. It also increases the rates of leaching that allows solvents to come into contact with bioactive compounds. Therefore, 1:10 (g/mL) of solvent ratio was chosen as the optimum ratio to maximise the speed of mass transfer^23,46,47^. Also, a high solvent ratio helps to maximise the extraction rate, minimise the use of latex and increase the percent of yield.

The TPC and antioxidant activity of the extracts from *F. carica* latex are mainly due to the presence of different active compounds. The highest result for specific cultivars may be due to the presence of more bioactive compounds than other cultivars. Maceration extraction was better than UAE for obtaining extracts from *F. carica* latex. In the case of *F. carica* latex, soaking and shaking help increase the amount of antioxidants and bioactive compounds obtained from the latex^30,44,45,48^. UAE was conducted by ultrasound but without shaking. The solvents used in soaking and shaking for maceration extraction also play a vital role^49^. During maceration, the tissues of *F. carica* latex are disintegrated first by shaking and heating. Finally, the desired bioactive compounds were diffused from the cell sap to the solvent and showed higher activity than UAE. However, most of the bioactive compounds found inside the cells cannot permeate the cell walls. Most of the water-soluble components with low molecular weights generally diffuse out of the cell when the tissue is treated, and its osmotic control is disrupted. An example is continuous shaking or heating to 60 °C. Even when an osmotic barrier is absent, the diffusion from tissues is often slow, especially with large molecules, such as proteins or gums^50–52^. Thus, the tissues of *F. carica* latex were disintegrated first by shaking and heating. Finally, the desired bioactive compounds were diffused from the cell sap to the solvent and showed high activity. Maceration may also work for extracting other non-antioxidant and polyphenolic compounds, which cannot be done via UAE.

Data showed that the difference between maceration and UAE was higher for TEAC than the percentage of inhibition. This result is due to the high range of values for TEAC. The SDs amongst the three replicates of the same cultivars are also significant for TEAC but not for the percentage of inhibition. ‘White Genoa’ showed the highest TPC and antioxidant activity, which might be due to the presence of more bioactive polyphenolic compounds than the other cultivars. Moreover, non-antioxidant compounds may also affect the antioxidant activity of *F. carica* latex^53,54^. The latex of ‘White Genoa’ is stickier and more viscous than those of other cultivars. Thus, the latex of this cultivar is more concentrated than those of others. The latex concentration may also affect the antioxidant activity of different cultivars. However, to the best our knowledge, information about the relationship between the physical properties of plant latex and antioxidant activity has not been reported yet.

Data from the FRAP assay indicated that the antioxidant capacities of the samples extracted via maceration and UAE were not significant for all cultivars. Thus, the antioxidant components in proportion to various cultivars cannot be isolated via FRAP assay. The antioxidants present in the *F. carica* latex exhibited reducing power by reducing Fe^3^ to Fe^2^. The values of the three different assays used to measure the antioxidant activity of *F. carica* latex varied. These differences are attributed to the varying reaction mechanisms of the assays. Moreover, the antioxidants from extracts have different abilities to mitigate peroxyl radicals and to reduce the ABTS^+^, DPPH free radical and ferric ion^55,56^. This phenomenon may be also due to their different properties, such as molecular size. ABTS radical is formed initially, whilst DPPH radical is a stabilised radical itself. They may also have different affinities against the compounds present in the sample. However, a positive correlation exists amongst the assays due to their similar redox reaction^57^.

Some authors also have reported that maceration extraction is more effective than other methods. Ethanolic extracts of *Psidium guajava* L. obtained via maceration showed the highest yield of phytoconstituents^44^. The antioxidant activity of methanolic extracts from *Garcinia atroviridis* obtained via maceration showed good results with a minimum EC_50_ value of 9.32 and 5.32 μg/mL for DPPH and ABTS assay, respectively^45^. The extracts of *Cosmos caudatus*^48^ and *M. oleifera*^30^ obtained via maceration exhibited the highest activity compared with others. The 70% ethanolic extract of *M. oleifera* obtained using maceration showed a minimum EC_50_ value of 62.94 μg/mL with DPPH and 51.50 mmol FeSO_4_ eqv/100 g of extract with FRAP assay. The DPPH values for squeezing, decoction and percolation were 367.32, 123.44 and 95.94 μg/mL, respectively. Maceration was used to extract the *F. carica* latex^11^ from Tunisian caprifig.

From the cluster analysis of 18 *F. carica* cultivars, the cultivar ‘White Genoa’ had the best antioxidant effect and can be used as a natural source of TPC and antioxidants. Correlation analysis revealed that DPPH and ABTS can be used to evaluate the antioxidant activity of *F. carica* extracts. Previously, Ajmol et al.^58^ reported that the fig leaf, peel and pulp contain 0.25, 0.19 and 0.04 g/100 g of TPC. In comparison with the activities of other *F. carica* parts^58–65^ and the latex of other plants^66–73^, *F. carica* latex showed good antioxidant activity, as well as TPC, and the DPPH, ABTS, FRAP and TPC values from some cultivars (‘White Genoa’, ‘B110’, ‘Alma’) are higher than those of previous studies.

## Conclusion

The extract from the ‘White Genoa’ latex obtained via maceration showed the highest antioxidant activity and TPC compared with that obtained via UAE. The latex of ‘B110’ and ‘Alma’ also showed good activities compared with ‘White Genoa’. Although ‘White Genoa’ showed the highest antioxidant activity and TPC, ‘B110’ and ‘Alma’ are also potential sources of TPC and natural antioxidants. The latex of these three *F. carica* L. cultivars could be a potential source of natural antioxidants and polyphenols. The estimation of total cost to isolate the antioxidant compounds from the latex of cultivar ‘White Genoa’ of *F. carica* commercially will help in the proper selection of technology for real-life applications. Developing a cost-effective natural extract with an efficacy similar to or better than that of the current *F. carica* cultivars could draw a substantial market share. The latex of *F. carica* cultivars with the highest activity can be subjected to in vitro and in vivo studies to consider their modes of action as a antioxidant. Also, these cultivars can be potential candidates for further phytochemical and pharmacological studies. However, further research should be carried out to determine the effects of the physical properties of fig latex (viscosity and water content), season, cultivation condition (fertiliser application and watering) and soil properties (physical and chemical properties) on the antioxidant activity of the reported fig cultivars.

## Supplementary information

Supplementary file1 (DOCX 32 kb)
