# Supplementary material for: In vitro antioxidant activity of Ficus carica L. latex from 18 different cultivars
Source: Sci Rep. 2020 Jul 2;10:10852. doi: 10.1038/s41598-020-67765-1 (PMC7331616; doi:10.1038/s41598-020-67765-1)
Supplement: Supplementary file 1 — Supplementary file1 (DOCX 32 kb) [file 41598_2020_67765_MOESM1_ESM.docx]

*In vitro* antioxidant activity of *Ficus carica* L. latex from 18 different cultivars

**M. Shahinuzzaman^1, 2, *^, Zahira Yaakob^1^, Farah Hannan Anuar^2^, Parul Akhtar^1^, N.H.A. Kadir^3^, A K Mahmud Hasan^4^, K. Sobayel^5^, Majid Nour^6^, Hatem Sindi^6^, Nowshad Amin^7^, K. Sopian^4^, Md. Akhtaruzzaman^4^, ^*^**

^1^ Department of Chemical and Process Engineering, Faculty of Engineering and Built Environment, Universiti Kebangsaan Malaysia, Bangi 43600, Selangor, Malaysia

^2^ School of Chemical Sciences and Food Technology, Faculty of Science and Technology, Universiti Kebangsaaan Malaysia, 43600 UKM Bangi, Selangor, Malaysia

^3^ School of Fundamental Science, Universiti Malaysia Terengganu, Terengganu, Malaysia.

^4^ Solar Energy Research Institute, Universiti Kebangsaan Malaysia, Bangi 43600, Selangor, Malaysia

^5^ Department of Electrical, Electronic and Systems Engineering, Faculty of Engineering and Built Environment, Universiti Kebangsaan Malaysia, Bangi 43600, Selangor, Malaysia.

^6^ Department of Electrical and Computer Engineering, Faculty of Engineering, King Abdulaziz University, Jeddah 21589, Saudi Arabia

^7^ Institute of Sustainable Energy, Universiti Tenaga Nasional (@The National Energy University), Jalan IKRAM-UNITEN, 43000 Kajang, Selangor, Malaysia

* Corresponding authors:

**M. Shahinuzzaman,** Email: shahinuzzaman@ukm.edu.my

**Md. Akhtaruzzaman**, Email: akhtar@ukm.edu.my; Tel.: +603-89118587

**Supplementary materials**

**Table S1:** Effect of solvent concentration with cultivar ‘B110’

| Solvent | DPPH (%) | | TPC (μg GAE/mL) | |
| --- | --- | --- | --- | --- |
|  | Maceration | Ultrasonic | Maceration | Ultrasonic |
| Methanol (CH_3_OH) 100% | 63.84 ± 1.04a | 53.65 ± 1.60a | 314.81 ± 6.23a | 289.11 ± 8.06a |
| Ethanol (C_2_H_5_OH) 100% | 51.12 ± 1.06c | 46.22 ±1.35b | 271.66 ± 7.12c | 243.85 ± 4.68c |
| Ethanol (C_2_H_5_OH) 75% | 58.82 ± 0. 8b | 51.3 ± 1.2a | 292.4 ± 4.61b | 259.21 ± 5.53b |
| Ethyl acetate (C_4_H_8_O_2_) 100% | 16.55 ± 0.93d | 14.05 ± 0.88c | 89.07 ± 2.11d | 71.48 ± 3.78d |
| n-hexane (C_6_H_14_) 100% | 7.43 ± 0.51e | 7.03 ± 0.17d | 61.26 ± 4.3e | 54.0 ± 3.05e |

Data are represented as the mean ± SD of three measurements. Different letters (a-e) for each column symbolise significant differences (p < 0.05) by mean of Tukey HSD test.

**Table S2:** Effect of latex to solvent ratio with cultivar ‘B110’ using maceration extraction process

| Latex to Solvent ratio (w/v) | DPPH scavenging capacity (%) | TPC (μg GAE/mL) |
| --- | --- | --- |
| 1:1 | 61.09 ± 1.35b | 287.5 ± 6.5b |
| 1:5 | 66.03 ± 1.88a | 306.62 ± 10.21a |
| 1:10 | 56.84 ± 1.51c | 291.16 ± 6.36b |
| 1:15 | 48.72 ± 1.80d | 208.22 ± 4.09d |

Data are represented as the mean ± SD of three measurements. Different letters (a-d) for each column symbolise significant differences (p < 0.05) by mean of Tukey HSD test.

**Table S3:** TPC of 18 *F. carica* cultivars latex using Folin-Ciocalteu assay

|  | Total phenolic content (µg GAE/mL) | |
| --- | --- | --- |
| *F. carica* cultivar | Maceration | Ultrasonication |
| Wuhan | 294.25 ± 11.23bcd | 260.21 ± 8.30bc |
| White Genoa | 311.83 ± 6.93a | 291.98 ± 13.40a |
| Masui Dauphine | 279.84 ± 10.62defg | 237.99 ± 9.01fghi |
| Violette Solise | 259.61 ± 5.68h | 248.90 ± 9.37cdef |
| Bourla Sotte | 268.70 ± 6.06gh | 266.17 ± 5.04b |
| Orphan | 277.18 ± 2.42efg | 253.55 ± 7.27bcd |
| Qing Pi | 293.95 ± 10.14bcde | 255.63 ± 9.86bcd |
| Lisa | 285.06 ± 11.61cdefg | 238.21 ± 9.10fghi |
| Longue d'Aout | 290.41 ± 5.88bcdef | 224.37 ± 7.95i |
| Brunswick | 299.00 ± 9.09abc | 253.55 ± 6.33bcd |
| Dow Law | 233.14 ± 12.25i | 243.24 ± 10.32defg |
| A-134 | 280.62 ± 14.72defg | 233.98 ± 8.66hi |
| Fen Chan Huang | 256.58 ± 12.12h | 260.01 ± 9.42bc |
| B110 | 303.14 ± 13.80ab | 254.80 ± 9.25bcd |
| B1011 | 276.47 ± 11.00fg | 252.08 ± 9.76bcde |
| A-132 | 256.68 ± 10.09h | 233.01 ± 7.49hi |
| Alma | 295.97 ± 4.10abcd | 235.30 ± 7.28ghi |
| Panachee | 284.76 ± 13.95cdefg | 256.66 ± 8.47bcd |

Data are represented as the mean ± SD of three measurements. Different letters (a-i) for each column symbolise significant differences (p < 0.05) by mean of Tukey HSD test.

**Table S4:** Antioxidant activity of 18 *F. carica* cultivars latex by DPPH assay

|  | Antioxidant activity (DPPH) | | | |
| --- | --- | --- | --- | --- |
|  | Maceration | | Ultrasonication | |
| *F. carica* cultivar | % of inhibition | TEAC (µg TE/mL) | % of inhibition | TEAC (µg TE/mL) |
| Wuhan | 23.54 ± 1.18l | 128.25 ±7.60l | 22.18 ± 1.66k | 119.50 ± 10.68k |
| White Genoa | 64.93 ± 2.00a | 394.17 ± 12.82a | 58.22 ± 1.78a | 351.08 ± 11.41a |
| Masui Dauphine | 44.87 ± 2.17ij | 259.08 ± 13.94ij | 36.90 ± 1.08hij | 207.00 ± 6.96hij |
| Violette Solise | 43.40 ± 1.97jk | 249.50 ± 12.69jk | 39.45 ± 1.41fg | 223.67 ± 9.04fg |
| Bourla Sotte | 53.47 ± 2.02e | 315.33 ± 13.01e | 35.50 ± 1.57ij | 197.83 ± 10.10ij |
| Orphan | 42.86 ± 1.69jk | 252.38 ± 10.87jk | 34.28 ± 1.61ij | 197.22 ± 10.32ij |
| Qing Pi | 20.82 ± 1.54l | 110.75 ± 9.92l | 18.16 ± 1.07k | 93.67 ± 6.88l |
| Lisa | 57.96 ± 2.04cd | 344.67 ± 13.14cd | 49.01 ± 1.95c | 286.17 ± 12.52c |
| Longue d'Aout | 56.72 ± 1.68d | 336.58 ± 10.78d | 53.98 ± 2.15b | 318.67 ± 13.83b |
| Brunswick | 49.59 ± 1.77gh | 289.92 ± 11.34gh | 45.32 ± 1.36d | 262.00 ± 8.75d |
| Dow Law | 47.80 ± 1.59hi | 278.25 ± 10.23hi | 39.12 ± 0.91fgh | 221.50 ± 5.85fgh |
| A-134 | 52.71 ± 1.17ef | 310.33 ± 7.53ef | 40.34 ± 2.10ef | 229.50 ± 13.52ef |
| Fen Chan Huang | 50.86 ± 2.19efg | 298.25 ± 14.09efg | 42.38 ± 0.74e | 242.79 ± 4.76e |
| B110 | 61.38 ± 1.75b | 367.00 ± 11.25b | 53.15 ± 1.17b | 313.25 ± 7.50b |
| B1011 | 49.85 ± 2.13fgh | 291.63 ± 13.69fgh | 38.81 ± 0.89fgh | 219.50 ± 5.73fgh |
| A-132 | 41.43 ± 1.87k | 236.58 ± 12.01k | 34.74 ± 1.57j | 192.83 ± 10.10j |
| Alma | 59.97 ± 2.15bc | 357.83 ± 13.83bc | 50.41 ± 2.14c | 295.33 ± 13.77c |
| Panachee | 41.76 ± 2.02jk | 245.33 ± 13.01jk | 37.22 ± 0.63ghi | 209.08 ± 4.02ghi |

Data are represented as the mean ± SD of three measurements. Different letters (a-l) for each column symbolise significant differences (p < 0.05) by mean of Tukey HSD test.

**Table S5:** Antioxidant activity of 18 *F. carica* cultivars latex by ABTS assay

| ***F. carica* cultivars** | Antioxidant activity (ABTS) | | | |
| --- | --- | --- | --- | --- |
|  | Maceration | | Ultrasonication | |
|  | % of inhibition | TEAC (µg TE/mL) | % of inhibition | TEAC (µg TE/mL) |
| Wuhan | 59.26 ± 1.48l | 245.03 ± 10.60l | 52.28 ± 0.56l | 226.10 ± 3.62l |
| White Genoa | 98.81 ± 0.34a | 528.78 ± 2.44a | 79.64 ± 1.69a | 414.55 ± 11.03a |
| Masui Dauphine | 74.88 ± 1.15gh | 357.15 ± 8.23gh | 66.23 ± 1.80gh | 317.07 ± 11.75gh |
| Violette Solise | 72.67 ± 1.84hi | 341.26 ± 13.22hi | 61.78 ± 2.32i | 288.07 ± 15.10i |
| Bourla Sotte | 81.22 ± 1.39e | 402.60 ± 10.00e | 69.94 ± 2.32e | 341.28 ± 15.12e |
| Orphan | 72.88 ± 0.93hi | 342.79 ± 6.64hi | 61.03 ± 1.62hi | 283.18 ± 10.55hi |
| Qing Pi | 53.47 ± 0.79m | 203.52 ± 5.70m | 42.60 ± 1.10m | 162.99 ± 7.16m |
| Lisa | 85.04 ± 1.46d | 429.97 ± 9.30d | 73.62 ± 1.71d | 365.27 ± 11.16d |
| Longue d'Aout | 87.07 ± 1.34d | 444.59 ± 9.58d | 75.97 ± 2.11d | 380.63 ± 13.73d |
| Brunswick | 75.31 ± 1.91g | 360.21 ± 13.74g | 59.78 ± 2.13g | 274.99 ± 13.88g |
| Dow Law | 71.62 ± 1.73ij | 333.73 ± 12.44ij | 52.54 ± 1.71ij | 227.83 ± 11.14ij |
| A-134 | 80.30 ± 1.15e | 396.04 ± 8.22e | 70.46 ± 1.15e | 344.66 ± 7.52e |
| Fen Chan Huang | 78.02 ± 1.69f | 379.64 ± 12.11f | 65.82 ± 1.83f | 314.39 ± 11.93f |
| B110 | 96.18 ± 1.13b | 509.90 ± 8.12b | 80.14 ± 2.19b | 407.83 ± 14.27b |
| B1011 | 80.93 ± 1.59e | 400.51 ± 11.42e | 66.48 ± 1.73e | 318.72 ± 11.31e |
| A-132 | 68.93 ± 0.81k | 314.44 ± 5.81k | 54.42 ± 1.51k | 240.08 ± 9.88k |
| Alma | 91.45 ± 1.66c | 475.98 ± 11.87c | 79.89 ± 1.58c | 406.20 ± 10.31c |
| Panachee | 69.43 ± 0.99jk | 318.05 ± 7.07jk | 59.54 ± 2.11jk | 273.46 ± 13.75jk |

Data are represented as the mean ± SD of three measurements. Different letters (a-l) for each column symbolise significant differences (p < 0.05) by mean of Tukey HSD test.

**Table S6:** Antioxidant activity of 18 *F. carica* cultivars latex by FRAP assay

| *F. carica* cultivars | Antioxidant activity (FRAP)  TEAC (mg TE/g) | |
| --- | --- | --- |
|  | Maceration | Ultrasonication |
| Wuhan | 15.63 ± 0.71hij | 14.21 ± 0.71ef |
| White Genoa | 26.14 ± 0.98a | 24.71 ± 0.80a |
| Masui Dauphine | 15.01 ± 0.55ijk | 13.58 ± 0.97fg |
| Violette Solise | 16.53 ± 0.43fgh | 14.96 ± 1.18def |
| Bourla Sotte | 18.05 ± 0.93cde | 16.15 ± 0.72cd |
| Orphan | 20.69 ± 0.93b | 19.24 ± 0.91b |
| Qing Pi | 18.74 ± 0.61cd | 17.31 ± 0.92c |
| Lisa | 17.34 ± 0.47defg | 14.96 ± 0.36def |
| Longue d'Aout | 18.88 ± 1.03c | 17.16 ± 0.84c |
| Brunswick | 14.15 ± 1.55kl | 12.24 ± 1.54gh |
| Dow Law | 13.92 ± 0.64kl | 12.55 ± 0.96gh |
| A-134 | 16.79 ± 1.25efgh | 14.41 ± 1.00ef |
| Fen Chan Huang | 15.92 ± 0.32hi | 14.62 ± 0.75ef |
| B110 | 21.19 ± 0.80b | 18.81 ± 0.86b |
| B1011 | 14.28 ± 1.02jkl | 12.43 ± 0.60gh |
| A-132 | 13.05 ± 0.31l | 11.25 ± 0.54h |
| Alma | 16.21 ± 0.91ghi | 14.31 ± 0.88ef |
| Panachee | 17.64 ± 0.64cdef | 15.26 ± 0.33de |

Data are represented as the mean ± SD of three measurements. Different letters (a-l) for each column symbolise significant differences (p < 0.05) by mean of Tukey HSD test.
